# Supplementary material for: Germline-restricted chromosome of songbirds has different centromere compared to regular chromosomes
Source: Heredity (Edinb). 2025 Jul 30;135(8):638–45. doi: 10.1038/s41437-025-00779-5 (PMC13545247; doi:10.1038/s41437-025-00779-5)
Supplement: Supplementary file 1 [file 41437_2025_779_MOESM1_ESM.pdf]

Supplementary Figures for

Germline-restricted chromosome of songbirds has different centromere compared to regular chromosomes

The document includes:

Supplementary Figure 1
Supplementary Figure 2
Supplementary Figure 3
Supplementary Figure 4
Supplementary Figure 5
Supplementary Figure 6
Supplementary Figure 7
Supplementary Figure 8
Supplementary Figure 9
Supplementary Figure 10
References

26

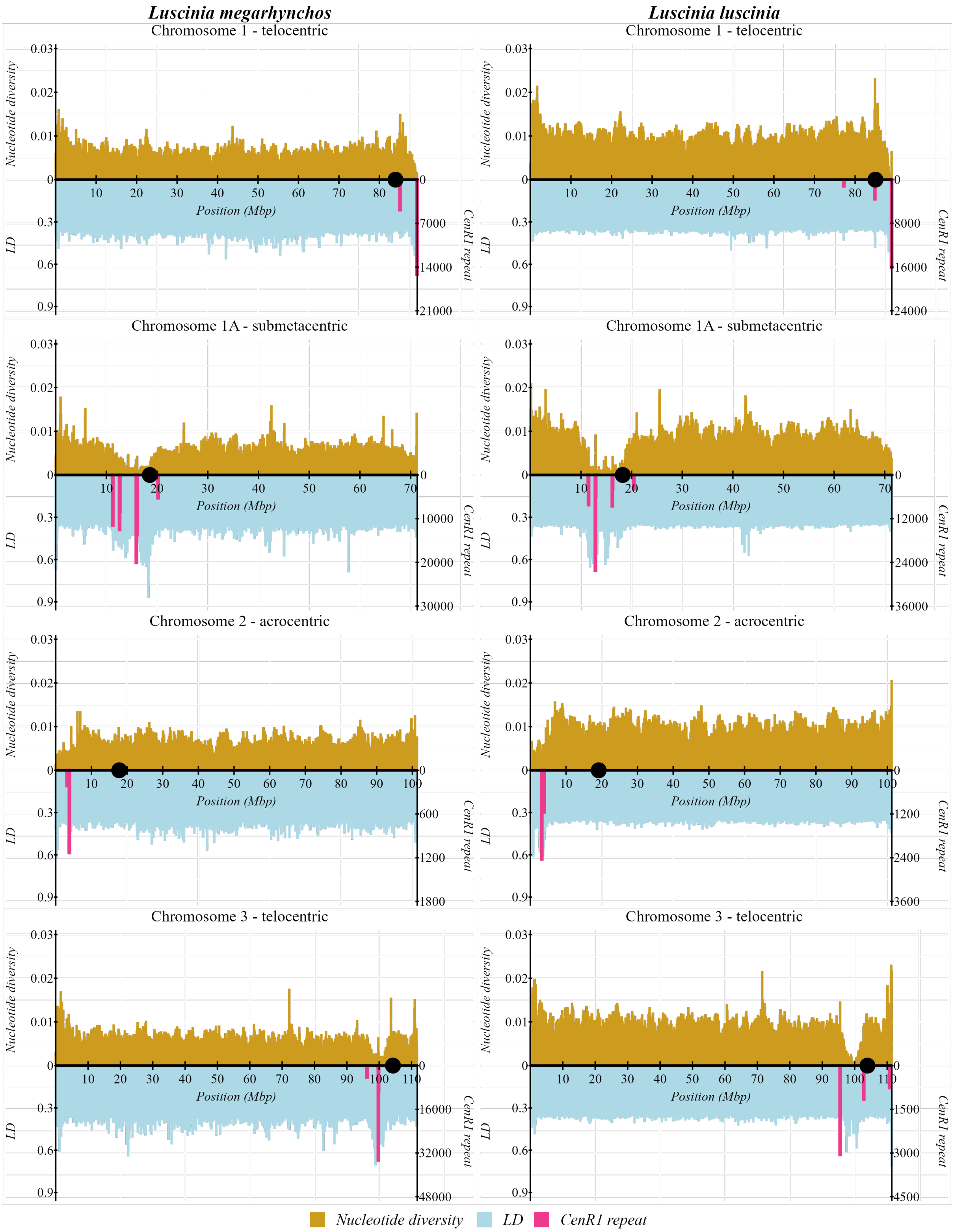

Luscinia megarhynchos
Luscinia luscinia
Chromosome 1 - telocentric
Chromosome 1A - submetacentric
Chromosome 2 - acrocentric
Chromosome 3 - telocentric
Nucleotide diversity
LD
CenR1 repeat
Position (Mbp)
Nucleotide diversity
LD
CenR1 repeat

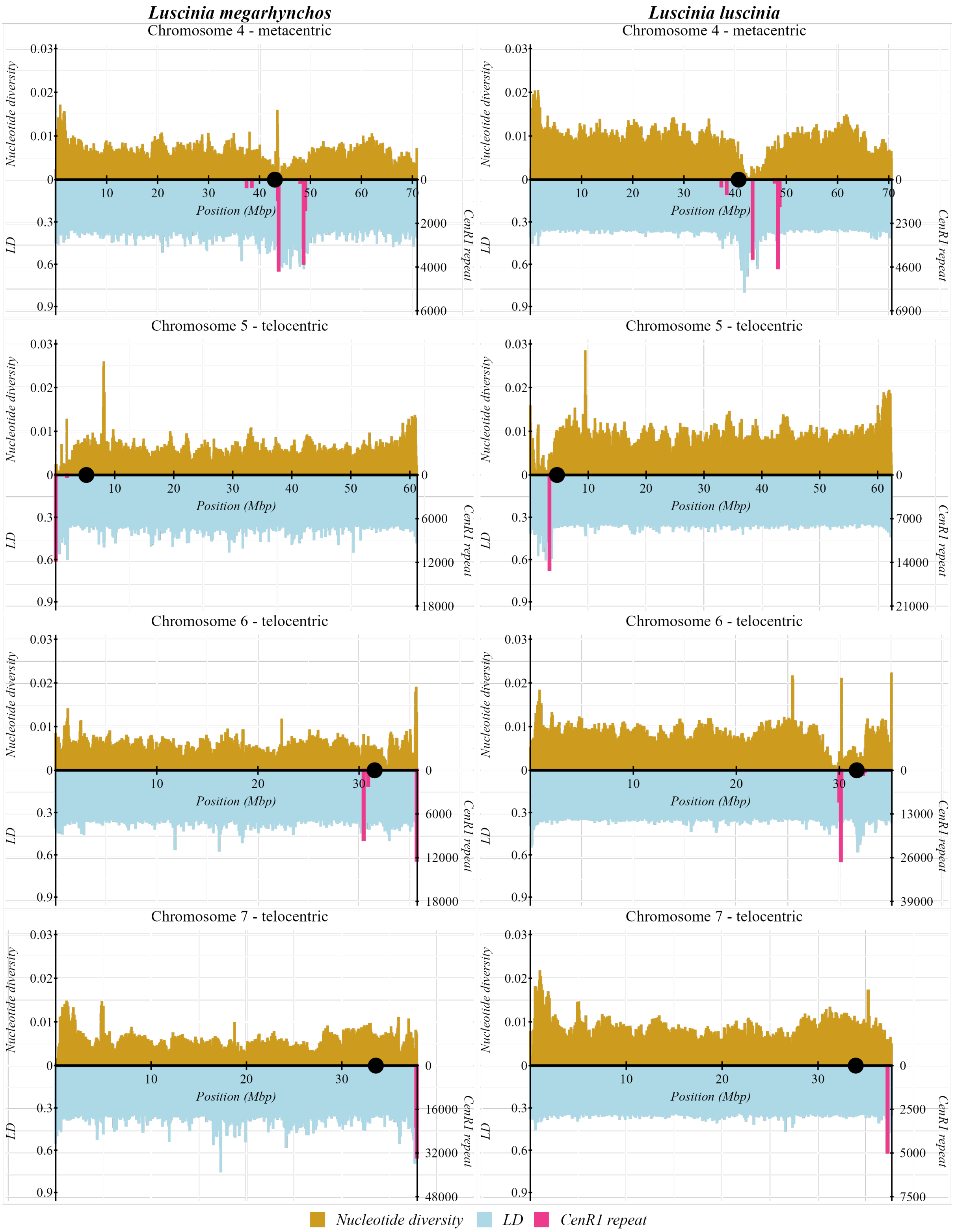
Luscinia megarhynchos
Luscinia luscinia
Chromosome 4 - metacentric
Chromosome 5 - telocentric
Chromosome 6 - telocentric
Chromosome 7 - telocentric
Nucleotide diversity
LD
Position (Mbp)
CenR1 repeat
Nucleotide diversity
LD
CenR1 repeat

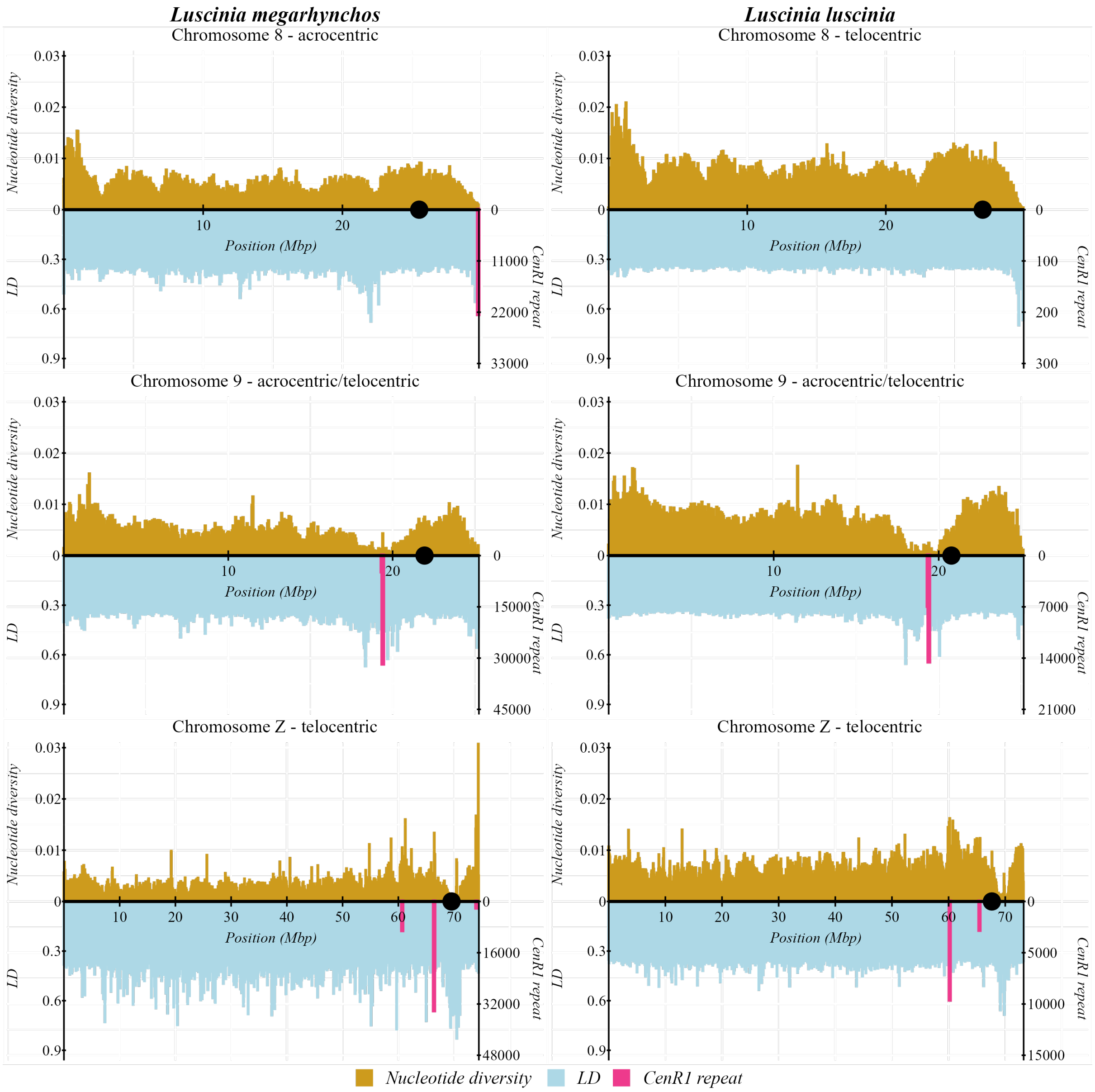

Supplementary Figure 1. Nucleotide diversity (π) (yellow), linkage disequilibrium (LD) (blue) and the cumulative length of the centromeric satellites *LmegCenR1* and *LlusCenR1* (magenta) in 50kb sliding windows along the nightingale chromosomes. The ten largest chromosomes and the Z chromosome, which we were able to homologize with the karyotype data (Poignet et al. 2021) based on the chromosome lengths are shown. Chromosome morphology classification (telocentric, acrocentric, submetacentric and metacentric) is based on the estimated position of centromere from cytogenetic data. This position shows the median arm ratio (Poignet et al. 2021) and is marked by black dots on individual

chromosomes. The estimated positions of centromeres from cytogenetic data roughly colocalize with the genomic regions of decreased nucleotide diversity and elevated linkage disequilibrium and with the regions containing *CenR1* repeats, although some discrepancies are present, likely caused by missing parts of our assemblies. The absence of *CenR1* repeats in the *Luscinia luscinia* assembled chromosome 8 is likely a consequence of an unassembled end of this chromosome. This is supported by the location of the *CenR1* peak in *Luscinia megarhynchos* at the very right end of the chromosome.

```
LmegCR1     TCTGCACGACGAATAACTATGTTGTAGCAAAGCTGGTTCTGGCATATCCTGCTTCATACC
LlusCR1     TCTGCACGACGAATAACTATGTTGTAGCTAAGCTGGTTCTGGCATATCCTGCTTCATACC
            **************************** *******************************

LmegCR1     ACTAGTCCCTGACAACCGGTATTCTGTGCAGAGCACCTCAGTGCAGTGTGGTTTCCCTAA
LlusCR1     ACTAGTCCCTGACAACCGGTATTCTGTGCAGAGCACCTCAGTGCAGTGTGGTTTCCTTAA
            ********************************************************.***

LmegCR1     TCGCGGTTTGTAGCAAGAAGCGATTTCTGTCTAAGGGAGGAGAAGTGCCGTCTACCCCTC
LlusCR1     TGGCGGTTTGTAGCAAGAAGCGATTTCAGGCTGAGGGAGGTGAAGTGCCGTCTACCCCTC
            * ************************* * **.******* *******************

LmegCR1     TCACAGAACACTCTGCTGGGAAGTTCTGCTACTGCCAAAACACGCCTGGCATGTGCACTT
LlusCR1     TCACAGAACACTCTGCTGGGAAGTTCTGCTACTGCCAAAACACGCCTGGCATGTGCACTT
            ************************************************************

LmegCR1     TTCTTCTAAAACCTCTCTACCATAGCAATATTCTCTGCTTCTTGAAAGCATACCATACAC
LlusCR1     TTCTTCTAAAACCTCTCTACCATAGCAATATTCTCTGCTTCTTGAAAGCATACCATACAC
            ************************************************************

LmegCR1     TCTTTCCACTATGCATAGCGAAGAGCACTGCAGTGCACCTAAAGCTAAAGCGAAAGTGTG
LlusCR1     TCTTTCCACTATGCATAGCGAAGAGCACTGCAGTGCACCTAAAGCTAAAGCGAAAGTGTG
            ************************************************************

LmegCR1     CACAGCACACCACAGATCTCACTTTGCACTACACCTAGAAATGGCTTCTCTGCATCACTG
LlusCR1     CACAGCACACCACAGATCTCACTTTGCACTACACCTAGAAATGGCTTCTCTGCATCACTG
            ************************************************************

LmegCR1     ATATCTAGCACAGAAA
LlusCR1     ATATCTAGCACAGAAA
            ****************
```

Supplementary Figure 2. Visualization of the *LmegCenR1* and *LlusCenR1* consensus sequence alignment using MAFFT v7.511 (Katoh et al. 2019). The position of the PCR primers used to generate the PCR probes are marked above the sequences by black arrows for *L. megarhynchos* and below the sequences by dashed arrows for *L. luscinia*. Sites targeted by oligoprobes are indicated in blue for *L. megarhynchos* and in red for *L. luscinia*.

```
                    ****    *  ****      identity
Consensus    (A/C)TTCGTTGGAAACGGGA
LmegCenR1rc       AGTGGTATGAAGCAGGA         65%
LlusCenR1rc       AGTGGTATGAAGCAGGA         65%
```

Supplementary Figure 3. Identification of a putative 17bp CENP-B box sequence in the *LmegCenR1* and *LlusCenR1* centromeric satellites with similarity to the mammalian consensus sequence. Nucleotides necessary for CENP-B binding in humans (Ohzeki et al. 2002) are marked by asterisks, nucleotides identical to the consensus are indicated in red, rc indicates reverse complementary sequence.

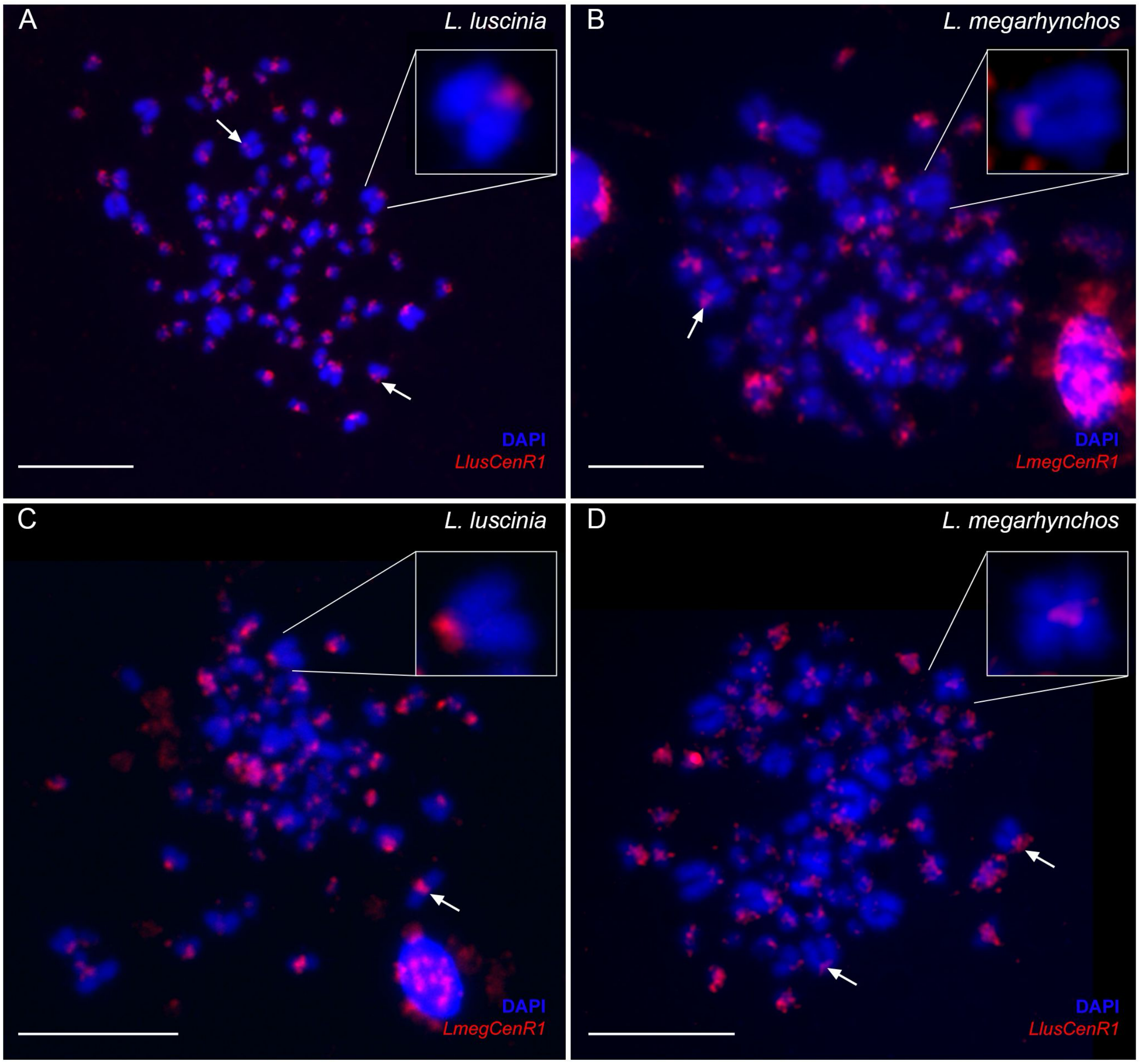

Supplementary Figure 4. FISH-based mapping of *LlusCenR1* repeat on *L. luscinia* (A) and *L. megarhynchos* (D) mitotic chromosomes and *LmegCenR1* repeat on *L. megarhynchos* (B) and *L. luscinia* (C) mitotic chromosomes. Insets show selected enlarged chromosomes. FISH signals are localized in centromeric regions of all chromosomes (indicated by arrows). Chromatin stained with DAPI. Scale bar = 10 µm.

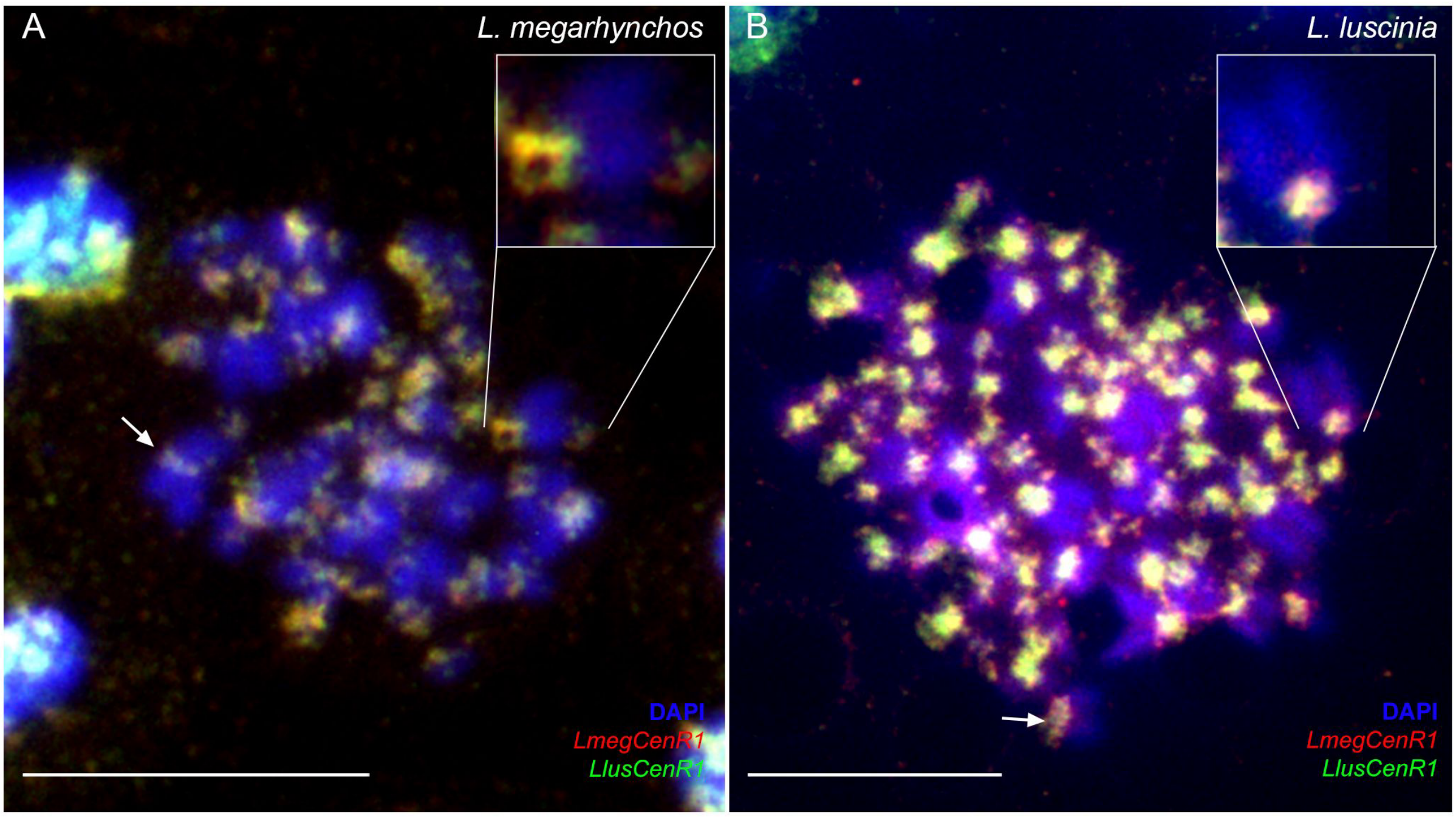

Supplementary Figure 5. Two-coloured FISH of *CenR1* probes on *L. megarhynchos* (A) and *L. luscinia* (B) mitotic chromosomes. *LlusCenR1* (green) and *LmegCenR1* (red) repeats are colocalized in the centromeric regions of all chromosomes in both species (A, B) (indicated by arrows). Chromatin stained with DAPI. Insets show selected enlarged chromosomes. Scale bar = 10 μm.

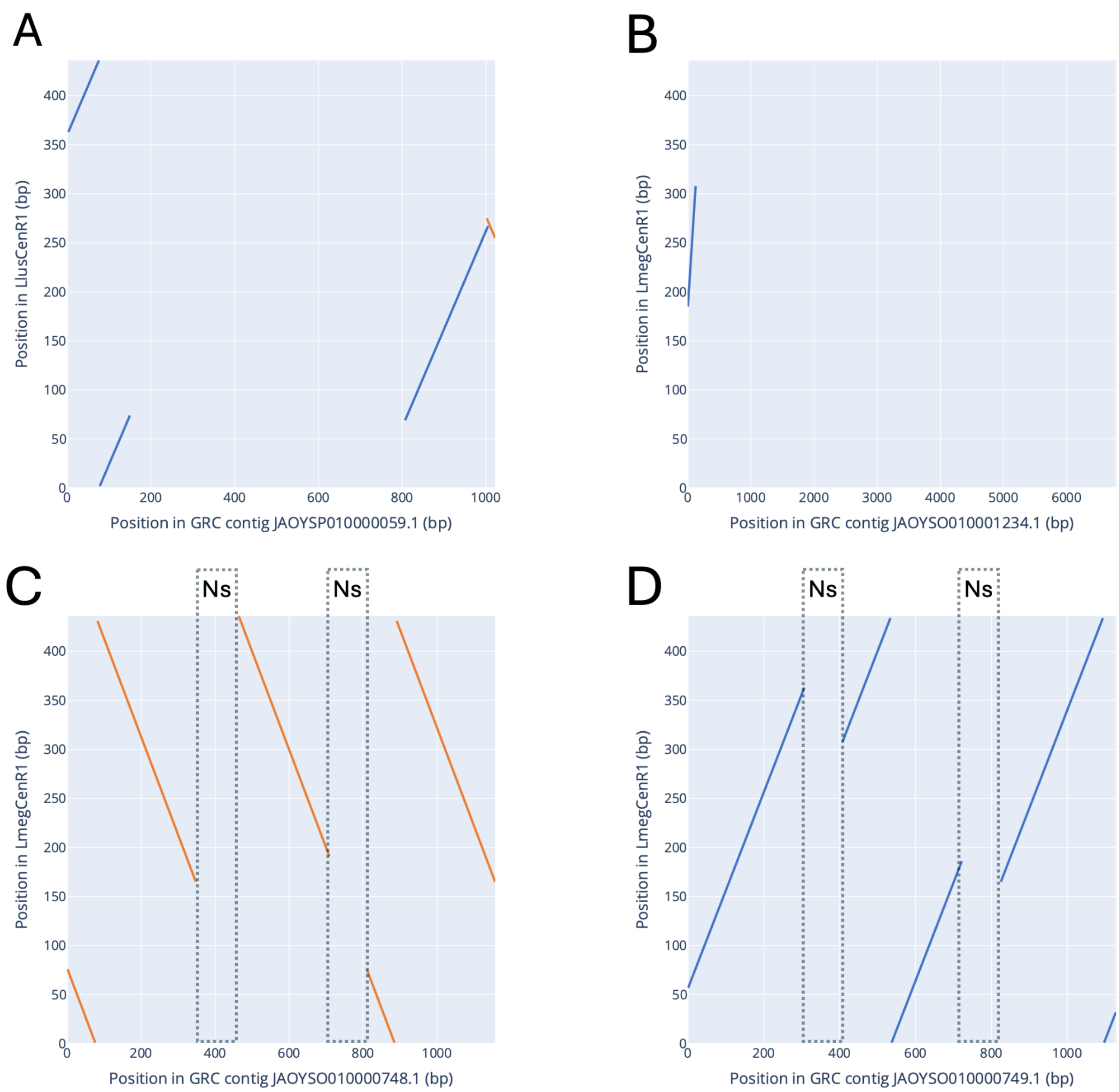

Supplementary Figure 6. Dot plots visualizing the BLASTN alignments of the *CenR1* sequences against one GRC scaffold in the thrush nightingale (A) and three GRC scaffolds in the common nightingale (B, C and D). The dot plots were generated using a python script from https://github.com/shelkmike/Dot_plot_like_in_BLAST. The gaps of undefined lengths (stretches of 100 Ns) in the contigs are indicated by the dashed line boxes.

```
Query = JAOYSP010000059.1 Luscinia luscinia isolate LL19 LL_GRC_scaffold_535, whole genome shotgun sequence
Sbjct = LlusCenR1

Score = 355 bits (393),  Expect = 7e-102
Identities = 198/199 (99%), Gaps = 0/199 (0%)
Strand=Plus/Plus
Query 807   AGTGCAAAGTGAGATCTGTGGTGTGCTGTGCACACTTTCGCTTTAGCTTTAGGTGCACTGCAGTGCTCTTCGCTATGCATAGTGGAAAGAGTGTATGGTA
            ||||||||||||||||||||||||||||||||||||||||||||||||||||||||||||||||||||||||||||||||||||||||||||||||||||
Sbjct 69    AGTGCAAAGTGAGATCTGTGGTGTGCTGTGCACACTTTCGCTTTAGCTTTAGGTGCACTGCAGTGCTCTTCGCTATGCATAGTGGAAAGAGTGTATGGTA

Query 907   TGCTTTCAAGAAGCAGAGAATATTGCTATGGTAGAGAGGTTTTAGAAGAAAAGTACACATGCCAGGCGTGTTTTGGCAGTAGCAGAACTTCCCAGCAGA
            |||||||||||||||||||||||||||||||||||||||||||||||||||||| |||||||||||||||||||||||||||||||||||||||||||||
Sbjct 169   TGCTTTCAAGAAGCAGAGAATATTGCTATGGTAGAGAGGTTTTAGAAGAAAAGTGCACATGCCAGGCGTGTTTTGGCAGTAGCAGAACTTCCCAGCAGA


Score = 130 bits (143),  Expect = 5e-34
Identities = 73/74 (99%), Gaps = 0/74 (0%)
Strand=Plus/Plus
Query 3     GTGCTCTGCACAGAACACCGGTTGTCAGGGACTAGTGGTATGAAGCAGGATATGCCAGAACCAGCTTAGCTACA
            ||||||||||||||| ||||||||||||||||||||||||||||||||||||||||||||||||||||||||||
Sbjct 363   GTGCTCTGCACAGAATACCGGTTGTCAGGGACTAGTGGTATGAAGCAGGATATGCCAGAACCAGCTTAGCTACA


Score = 128 bits (141),  Expect = 2e-33
Identities = 72/73 (99%), Gaps = 0/73 (0%)
Strand=Plus/Plus
Query 78    CATAGTTATTTGTCGTGCAGATTTCTGTGCTAGATATCAGTGATGCAGAGAAGCCATTTCTAGGTGTAGTGCA
            |||||||||| ||||||||||||||||||||||||||||||||||||||||||||||||||||||||||||||
Sbjct 2     CATAGTTATTCGTCGTGCAGATTTCTGTGCTAGATATCAGTGATGCAGAGAAGCCATTTCTAGGTGTAGTGCA


Score = 39.2 bits (42),  Expect = 7e-07
Identities = 21/21 (100%), Gaps = 0/21 (0%)
Strand=Plus/Minus
Query 1002  CAGAACACTCTGCTGGGAAGT
            |||||||||||||||||||||
Sbjct 275   CAGAACACTCTGCTGGGAAGT
```

Supplementary Figure 7. BLASTN Sequence alignment of the *LlusCenR1* and the thrush nightingale GRC scaffold NCBI accession number JAOYSP010000059.1.

```
Query = JAOYSO010001234.1 Luscinia megarhynchos isolate LM17 LM_GRC_scaffold_13661, whole genome shotgun sequence
Sbjct = LmegCenR1

Score = 206 bits (228),  Expect = 2e-56
Identities = 120/124 (97%), Gaps = 0/124 (0%)
Strand=Plus/Plus
Query 1     AGAACACTCTGCTGGGAAGTTCTGCTACCGCCAAAACACGCCTGGCACGTGCACTTTTCTTCTAAAACCTCTCTACCATAGCAATATTCTCTGCTTTTTG
            |||||||||||||||||||||||||||| |||||||||||||||||| |||||||||||||||||||||||||||||||||||||||||||||||| |||
Sbjct 185   AGAACACTCTGCTGGGAAGTTCTGCTACTGCCAAAACACGCCTGGCATGTGCACTTTTCTTCTAAAACCTCTCTACCATAGCAATATTCTCTGCTTCTTG

Query 101   AAAGCATACCATACACACTTTCCA
            |||||||||||||||| |||||||
Sbjct 285   AAAGCATACCATACACTCTTTCCA
```

Supplementary Figure 8. BLASTN Sequence alignment of the *LmegCenR1* and the common nightingale GRC scaffold NCBI accession number JAOYSO010001234.1.

```
Query = JAOYSO010000748.1 Luscinia megarhynchos isolate LM17 LM_GRC_scaffold_12425, whole genome shotgun sequence
Sbjct = LmegCenR1

Score = 419 bits (464),  Expect = 2e-121
Identities = 253/267 (95%), Gaps = 0/267 (0%)
Strand=Plus/Minus
Query 891   GTGGTAGATATCACTTGTGCAGAGAAGCCCTTTCTGGGTGTAGTGCAAAGTGAGATCTGTGGTGTGCTGTGCACACTTTCGCTTTAGCTTTAGGTGCACT
            ||| ||||||||| |  |||||||||||| ||||| ||||||||||||||||||||||||||||||||||||||||||||||||||||||||||||||||
Sbjct 431   GTGCTAGATATCAGTGATGCAGAGAAGCCATTTCTAGGTGTAGTGCAAAGTGAGATCTGTGGTGTGCTGTGCACACTTTCGCTTTAGCTTTAGGTGCACT

Query 991   GCAGTGCTCTTCGCTATGCATAGTGGAAAGATTGCATGGTAGGCTTTCAGGAAGCAGGAAGTATTGCTATGGTAGAGAGGTTTTAGAAGAAAAGTGCACA
            ||||||||||||||||||||||||||||||| || |||||| ||||||| |||||||  | |||||||||||||||||||||||||||||||||||||||
Sbjct 531   GCAGTGCTCTTCGCTATGCATAGTGGAAAGAGTGTATGGTATGCTTTCAAGAAGCAGAGAATATTGCTATGGTAGAGAGGTTTTAGAAGAAAAGTGCACA

Query 1091  TGCCAGGCGTGTTTTGGCAGGAGCAGAACTTCCCAGCAGAGTGTTCTGTGAGAGGGGTAGACGGCAC
            |||||||||||||||||||| ||||||||||||||||||||||||||||||||||||||||||||||
Sbjct 631   TGCCAGGCGTGTTTTGGCAGTAGCAGAACTTCCCAGCAGAGTGTTCTGTGAGAGGGGTAGACGGCAC


Score = 419 bits (464),  Expect = 2e-121
Identities = 253/267 (95%), Gaps = 0/267 (0%)
Strand=Plus/Minus
Query 82    GTGGTAGATATCACTTGTGCAGAGAAGCCCTTTCTGGGTGTAGTGCAAAGTGAGATCTGTGGTGTGCTGTGCACACTTTCGCTTTAGCTTTAGGTGCACT
            ||| ||||||||| |  |||||||||||| ||||| ||||||||||||||||||||||||||||||||||||||||||||||||||||||||||||||||
Sbjct 431   GTGCTAGATATCAGTGATGCAGAGAAGCCATTTCTAGGTGTAGTGCAAAGTGAGATCTGTGGTGTGCTGTGCACACTTTCGCTTTAGCTTTAGGTGCACT

Query 182   GCAGTGCTCTTCGCTATGCATAGTGGAAAGATTGCATGGTAGGCTTTCAGGAAGCAGGAAGTATTGCTATGGTAGAGAGGTTTTAGAAGAAAAGTGCACA
            ||||||||||||||||||||||||||||||| || |||||| ||||||| |||||||  | |||||||||||||||||||||||||||||||||||||||
Sbjct 531   GCAGTGCTCTTCGCTATGCATAGTGGAAAGAGTGTATGGTATGCTTTCAAGAAGCAGAGAATATTGCTATGGTAGAGAGGTTTTAGAAGAAAAGTGCACA

Query 282   TGCCAGGCGTGTTTTGGCAGGAGCAGAACTTCCCAGCAGAGTGTTCTGTGAGAGGGGTAGACGGCAC
            |||||||||||||||||||| ||||||||||||||||||||||||||||||||||||||||||||||
Sbjct 631   TGCCAGGCGTGTTTTGGCAGTAGCAGAACTTCCCAGCAGAGTGTTCTGTGAGAGGGGTAGACGGCAC


Score = 372 bits (412),  Expect = 3e-107
Identities = 230/246 (93%), Gaps = 0/246 (0%)
Strand=Plus/Minus
Query 464   TTTCTGTGGTAGATATGAATAATGCAGAGAAGCCATTTCTGGGTGTAGTGCAAAGTGAGATCTGTGGTGTGCTGTGCACACTTTCGCTTTAGCTTTAGGT
            |||||||| ||||||| | | ||||||||||||||||||| |||||||||||||||||||||||||||||||||||||||||||||||||||||||||||
Sbjct 436   TTTCTGTGCTAGATATCAGTGATGCAGAGAAGCCATTTCTAGGTGTAGTGCAAAGTGAGATCTGTGGTGTGCTGTGCACACTTTCGCTTTAGCTTTAGGT

Query 564   GCACTGCAGTGCTCTTCAATATGCAGAGTTGAAATTGTGTATGGTATGCTTTCAAGAAGCAGAGAATATTGGTATGGGAGAGAGGTTTGAGAAGAAAACT
            |||||||||||||||||  |||||| ||| ||||  ||||||||||||||||||||||||||||||||||| ||||| |||||||||| ||||||||| |
Sbjct 536   GCACTGCAGTGCTCTTCGCTATGCATAGTGGAAAGAGTGTATGGTATGCTTTCAAGAAGCAGAGAATATTGCTATGGTAGAGAGGTTTTAGAAGAAAAGT

Query 664   GCACATGCCAGGCTTGTTTTGGCAGTAGCAGAACTTCCCAGCAGAG
            ||||||||||||| ||||||||||||||||||||||||||||||||
Sbjct 636   GCACATGCCAGGCGTGTTTTGGCAGTAGCAGAACTTCCCAGCAGAG


Score = 102 bits (112),  Expect = 8e-26
Identities = 68/76 (89%), Gaps = 0/76 (0%)
Strand=Plus/Minus
Query 810   GTTGTCAGGGACTAGTGGTATGAAGCAGGGTATGCCAGAACCAGCATCTGTACAACATAGTTATTTGTCACGCAGA
            ||||||||||||||||||||||||||||| ||||||||||||||| |   ||||||||||||||| |||  |||||
Sbjct 76    GTTGTCAGGGACTAGTGGTATGAAGCAGGATATGCCAGAACCAGCTTTGCTACAACATAGTTATTCGTCGTGCAGA


Score = 102 bits (112),  Expect = 8e-26
Identities = 68/76 (89%), Gaps = 0/76 (0%)
Strand=Plus/Minus
Query 1     GTTGTCAGGGACTAGTGGTATGAAGCAGGGTATGCCAGAACCAGCATCTGTACAACATAGTTATTTGTCACGCAGA
            ||||||||||||||||||||||||||||| ||||||||||||||| |   ||||||||||||||| |||  |||||
Sbjct 76    GTTGTCAGGGACTAGTGGTATGAAGCAGGATATGCCAGAACCAGCTTTGCTACAACATAGTTATTCGTCGTGCAGA
```

Supplementary Figure 9. BLASTN Sequence alignment of the *LmegCenR1* and the common nightingale GRC scaffold NCBI accession number JAOYSO010000748.1.

```
Query = JAOYSO010000749.1 Luscinia megarhynchos isolate LM17 LM_GRC_scaffold_12426, whole genome shotgun sequence
Sbjct = LmegCenR1

Score = 446 bits (494),  Expect = 2e-129
Identities = 283/307 (92%), Gaps = 0/307 (0%)
Strand=Plus/Plus
Query 1     TACCACTAGTCCCTGACAACCGGTATTCTGTGCAGAGCACCTCAGTGCAGCGCGGTTTCTTCAATCGCGCTTTGTACCAAGCCCCGATTTTTCTCTAAGG
            |||||||||||||||||||||||||||||||||||||||||||||||||| | ||||||   ||||||| |||||| ||||   |||||| | |||||||
Sbjct 57    TACCACTAGTCCCTGACAACCGGTATTCTGTGCAGAGCACCTCAGTGCAGTGTGGTTTCCCTAATCGCGGTTTGTAGCAAGAAGCGATTTCTGTCTAAGG

Query 101   GGGGTGAGGTGCCGTCTACCCCTCTCACAGAGCGCTCTGCTGGGAAGTTCTGCTACTGCCAAAACACGCCTGGCATGTGCACTTTTCTTCTAGAACCTCT
            | || || ||||||||||||||||||||||| | |||||||||||||||||||||||||||||||||||||||||||||||||||||||||| |||||||
Sbjct 157   GAGGAGAAGTGCCGTCTACCCCTCTCACAGAACACTCTGCTGGGAAGTTCTGCTACTGCCAAAACACGCCTGGCATGTGCACTTTTCTTCTAAAACCTCT

Query 201   CTACCATAGCAATATTCTCTGCTGCTTGAAAGCATACCATACACTCTTTCAACTATGCATAGTGAAGAGCACTGCAGTGCACCTGAAGCCAAAGCGAAAG
            ||||||||||||||||||||||| |||||||||||||||||||||||||| ||||||||||| ||||||||||||||||||||| |||| ||||||||||
Sbjct 257   CTACCATAGCAATATTCTCTGCTTCTTGAAAGCATACCATACACTCTTTCCACTATGCATAGCGAAGAGCACTGCAGTGCACCTAAAGCTAAAGCGAAAG

Query 301   CGTGCAC
             ||||||
Sbjct 357   TGTGCAC


Score = 429 bits (475),  Expect = 4e-124
Identities = 257/270 (95%), Gaps = 0/270 (0%)
Strand=Plus/Plus
Query 825   GTGCCGTCTACCCCTCTCACAGAGCACTCTGCTGGGAAGTTCTGCTACTGCCAAAACACGCCTGGCATGTGCACTTTTCTTCTAAAACCTCTCTACCATA
            ||||||||||||||||||||||| ||||||||||||||||||||||||||||||||||||||||||||||||||||||||||||||||||||||||||||
Sbjct 165   GTGCCGTCTACCCCTCTCACAGAACACTCTGCTGGGAAGTTCTGCTACTGCCAAAACACGCCTGGCATGTGCACTTTTCTTCTAAAACCTCTCTACCATA

Query 925   GCAATATTCTCTGCTGCTTGAAAGCATACCATACACTCTTTCAACTATGCATAGCGAAGAGCACTTCAGTGCACCTAAAGCCAAAGCCAAAGCGTGCACA
            ||||||||||||||| |||||||||||||||||||||||||| |||||||||||||||||||||| ||||||||||||||| ||||| |||| |||||||
Sbjct 265   GCAATATTCTCTGCTTCTTGAAAGCATACCATACACTCTTTCCACTATGCATAGCGAAGAGCACTGCAGTGCACCTAAAGCTAAAGCGAAAGTGTGCACA

Query 1025  GCACAACACAGATCTCACTTTACACTACACCTAGAAATGACTTCTCTGCATCACGGGTATCTACCACAGA
            ||||| ||||||||||||||| ||||||||||||||||| |||||||||||||| | |||||| ||||||
Sbjct 365   GCACACCACAGATCTCACTTTGCACTACACCTAGAAATGGCTTCTCTGCATCACTGATATCTAGCACAGA


Score = 269 bits (297),  Expect = 9e-76
Identities = 171/186 (92%), Gaps = 0/186 (0%)
Strand=Plus/Plus
Query 537   TCTGCGCGACGAATAACTATCTTGTAGCAAATCTGGCTCTGGCATATCCTGCTTCATACCACTAGTCCCTGACAACCGGTATTCTGTGCAGAGCACCTCA
            ||||| |||||||||||||| |||||||||| |||| |||||||||||||||||||||||||||||||||||||||||||||||||||||||||||||||
Sbjct 1     TCTGCACGACGAATAACTATGTTGTAGCAAAGCTGGTTCTGGCATATCCTGCTTCATACCACTAGTCCCTGACAACCGGTATTCTGTGCAGAGCACCTCA

Query 637   GTGCAGTGTGGTTTCTTCAATCGCGCTTTGTACCAAGCCCTGATTTCTCTCTAAGGGAGGTGAAGTGCCGTCTACCCCTCTCACAG
            |||||||||||||||   ||||||| |||||| ||||    ||||||| ||||||||||| |||||||||||||||||||||||||
Sbjct 101   GTGCAGTGTGGTTTCCCTAATCGCGGTTTGTAGCAAGAAGCGATTTCTGTCTAAGGGAGGAGAAGTGCCGTCTACCCCTCTCACAG


Score = 180 bits (199),  Expect = 3e-49
Identities = 116/127 (91%), Gaps = 0/127 (0%)
Strand=Plus/Plus
Query 408   ACTATGCATAGTGAAGAGCACTGCAGTGCACCTGAAGCCAAAGCGAAAGCGTGCCCCGCACAGCACAGATCTCACTTTGCACTACACCTAGAAATGACTT
            ||||||||||| ||||||||||||||||||||| |||| |||||||||| |||| | ||||| ||||||||||||||||||||||||||||||||| |||
Sbjct 308   ACTATGCATAGCGAAGAGCACTGCAGTGCACCTAAAGCTAAAGCGAAAGTGTGCACAGCACACCACAGATCTCACTTTGCACTACACCTAGAAATGGCTT

Query 508   CTCTGCATCACGGATATCTTCCACAGA
            ||||||||||| |||||||  ||||||
Sbjct 408   CTCTGCATCACTGATATCTAGCACAGA


Score = 54.5 bits (59),  Expect = 3e-11
Identities = 31/32 (97%), Gaps = 0/32 (0%)
Strand=Plus/Plus
Query 1097  TCTGCGCGACGAATAACTATGTTGTAGCAAAG
            ||||| ||||||||||||||||||||||||||
Sbjct 1     TCTGCACGACGAATAACTATGTTGTAGCAAAG
```

Supplementary Figure 10. BLASTN Sequence alignment of the *LmegCenR1* and the common nightingale GRC scaffold NCBI accession number JAOYSO010000749.1.

References

Katoh K, Rozewicki J, Yamada KD (2019) MAFFT online service: multiple sequence alignment, interactive sequence choice and visualization. Brief Bioinform 20(4):1160–1166.

Ohzeki J, Nakano M, Okada T, Masumoto H (2002) CENP-B box is required for de novo centromere chromatin assembly on human alphoid DNA. J Cell Biol 159(5): 765.

Poignet M, Johnson Pokorná M, Altmanová M, Majtánová Z, Dedukh D, Albrecht T, et al. (2021) Comparison of Karyotypes in Two Hybridizing Passerine Species: Conserved Chromosomal Structure but Divergence in Centromeric Repeats. Front Genet 12: 768987.
