# Supplementary material for: Germline-restricted chromosome of songbirds has different centromere compared to regular chromosomes
Source: Heredity (Edinb). 2025 Jul 30;135(8):638–45. doi: 10.1038/s41437-025-00779-5 (PMC13545247; doi:10.1038/s41437-025-00779-5)
Supplement: Supplementary file 2 [file 41437_2025_779_MOESM2_ESM.pdf]

Supplementary Tables for

Germline-restricted chromosome of songbirds has different centromere compared to regular chromosomes

The document includes:

Supplementary Table 1
Supplementary Table 2

Supplementary Table 1. RepeatExplorer2/TAREAN output for five tandem repeats identified using 200,000 paired-end reads from *Luscinia megarhynchos*

Putative satellites (high confidence)

Cluster	Proportion[%]	Proportion adjusted[%]	Number of reads	Satellite probability	Consensus length	Consensus	Connected component index C	Pair completeness index P	TAREAN k-mer coverage	|V|	|E|	PBS score
CL1	6.40	6.40	14415	0.986	436	TCTGCACGACGAATAACTATGTTGTAGCAAAGCTGGTTCTGGCATATCCTGCTTCATACCACTAGTCCCTGACAACCGGT<br>ATTCTGTGCAGAGCACCTCAGTGCAGTGTGGTTTCCCTAATCGCGGTTTGTAGCAAGAAGCGATTTCTGTCTAAGGGAGG<br>AGAAGTGCCGTCTACCCCTCTCACAGAACACTCTGCTGGGAAGTTCTGCTACTGCCAAAACACGCCTGGCATGTGCACTT<br>TTCTTCTAAAACCTCTCTACCATAGCAATATTCTCTGCTTCTTGAAAGCATACCATACACTCTTTCCACTATGCATAGCG<br>AAGAGCACTGCAGTGCACCTAAAGCTAAAGCGAAAGTGTGCACAGCACACCACAGATCTCACTTTGCACTACACCTAGAA<br>ATGGCTTCTCTGCATCACTGATATCTAGCACAGAAA	0.996	0.966	0.842	10123	19999088	0.000
CL9	0.19	0.19	437	0.846	495	ATGTTTTCCCCAATAATGCTTGGAATTTTGACCGGTTTGGGCTGTTTCAGTGTCGATTTTAGGACTATTTCTGGCCATTT<br>TCATATGGATTTTGAGGGGATTTTTTGTTTCTTAGTGCCAATTTGTTAGGCTGATTTTCCCTGGATTTTTCCTGATGCTT<br>TCCCCAGCAGTCCTTGGAGTTTTGGCCGAGTTTACGTGTTCTAGTATGGATTTCAGGAGTCTTTGGGCCATTGTTCGTGT<br>CATTTCCACACTTTTTGGGCCATTTTATGTCTCATTTCCTCTGGATGTTTTCTCCGCTCATTTCCCCAGTTTTTCCTCAG<br>AAATTCCAGGAATCTCACTGGATTTTGGGGCCATGTTTGGCTTTTTAGTGCAGATGTTAGGTGTTTTTTCTGGCCATTAG<br>GGTGCAGTTTCTGGGGGATTTCTGTGTTTCTTAGTGGGAATTTTTAGGCATTTTCCTGGGTTGCTAAGGTTCAATTTATG<br>CTGGATTTTTTCCTG	0.947	0.917	0.304	437	7843	0.279
CL12	0.12	0.12	273	0.737	1309	ATGAATGTGGGGGCGGGATTTTGTTTCACAGGTTAACGGGCCACTATTTGAGTCCAGTGCCCTGGGCGTGAGAATTAAGA<br>CCCCTGCTAAAATGAGAAACGGATGAATATTTCTCCCAGATGCAAGGTGATATCATTTAGGTCTATGTCGGCCTGCAGAG<br>TGACAAAGGCCTTCTCCAAGCTGGTATCGGGAAACAGGGAGGGTGAGTACTTTTTCAAAGAGAAATAAGGCCATTATTTG<br>TGTCCAGGGCCCTGTGGCTGACACCGGGCTTCTGCATGCTGAAATTCTGAACGGATCAGCATTTCTCCCAGACACAAACT<br>GACACCACTTAGAACCCTGGCTGCTTTTTGAGTGCCAAAAGCCTCCTGTGAATAGACATTACAAAAGTGGTGAAGAGAGC<br>TGTATTTGACAGAGAAATGGGCTCGTTTCTGTCCATTGCCCAGTGAATGAAACGGGGCTTCTGCCTGCTAAAATTCCAAA<br>CAGTTCAGGATGTCTCACACACAATCTGGCAACACTTAGAACCCTGGCTGTTCTTTGAATTCCAAACACCCGCTCTAAAC<br>TGACATTCCAAAAGTGGTGAAGTGAGCTGTATTTCACAGAGAAATAGGCCCCTGTTTGTGTCCATTACCCAATGAATGAA<br>GTGAGGCTTCTGCCTGCTAAAACTCCAAACTGTTTGGAAATCTTGCAGATAGAAACGGGCACCATTAGAACCCTGGCTGT<br>TCTTTGGCTGACATCACCCAGTCTAAACTGACCTTATGAATGTGGGGACGGGGTTTTTGTTTCGCAGGTGACAGGGCCAC<br>TATTCATGTCCAGTGCCCCGGGCCTGAGAATTAAGTCCGCCTGCTAAAATGAGAAACAAATCAATATTTCTCCCAGATGC<br>AAGCTGCCATCACTTACAGCTATGTCTGCCTGCAGAGTGACAAGGGCCTCCTCCAGACTGGTATCGGGAAACTGGGAGGG<br>TGAGTATTTTTTTCAAAGTGAAAAGAGCCAATGTTGTTATCCATGGCCCTGTGCCTGAGACTGGGATTCTACACGGTAAA<br>ATTCTAAACCGAACAGTATTTCTCCCAGACTAAAGCTGCCACCACTTATAGCCCTGGAGGTCTATCCTGTGCCAAAAGCC<br>TCCTCCAAATCGACATTAAAGAAGTGGCGAAGTGAGCTCTATTTCACAGAGAAATAGGTCCCTGTGCCTGCGACTGGGCC<br>CTGTGCCTGCTAAAATTCCAAACTCTTAGGGAAGTCTCGCAGATACAGACTGGCACCATTAAAACCCTGGCTGTTCTTTG<br>AATGACATCAACCAGTCTTAACTGACCTT	0.941	0.923	0.838	273	4564	0.478

Putative satellites (low confidence)

Cluster	Proportion[%]	Proportion adjusted[%]	Number of reads	Satellite probability	Consensus length	Consensus	Connected component index C	Pair completeness index P	TAREAN k-mer coverage	|V|	|E|	PBS score
CL6	0.320	0.320	717	0.035	2643	CGGAGCAGTCTCTGGTGGGGTCTCCGCGCCCTTCGTGGCCGGGCAGGCGGCACAGAGCGGATCTCCGCCCGCTCCGTGGG<br>TCTCCTCCCGGTGCGCTGCTCGGGGCTCGTCTCCCAGGGACCCCTCCTCCTCTTTCCGCCGCCGTCTCTGCTGAGGGCCC<br>GAGCCAGCGGGGCTCTTTATATACCCAGACCAAGCCCCGCCCCTTTCAGGCACAGCCAATAGGAGCCCAGGAACAAGCGT<br>GGGGGCGGAGCACCCAGCGTTGCTCATTGTGATGTCACAAAGAGGAGGCGTGGCCACAGCGTGACCGAGCGTTCTGATTG<br>GTCGGAAGGAGAAAGAGCAGAAAAGGGGCGTGGCTCAGGCCCGGGGTGGGAGGGGCAGGTGTGAGGGCGGGGTGGGGGCG<br>GGGCCAGGGTCGCACCGGCATTTTGGGAGCAGGTCCGGGCGCTCCCGGTCCCTCCACAGCTCTCCTGGGCCCCTTCAGGC<br>ACTGCAAGAAGCAGAGGAAGGGGTCTCTGGGCGTCCCAGCCCCATTCCAGGAGTCGACAAGTGTCGACAGCGTGACGAGA<br>AGTTGCAACACTTATGGCTACAAGGACTTTTAAGGATTTATTCACCAATAACACACTGCCAACAAAGAGATTTATGTTTT<br>TAATGCAATACTAAAATTTTGTCTAATTGCAGTGTGAACTTTCTTAGCCAATCCTGTTCTGGCACACAGACCTAGTCTAA<br>GACTTTTTGTCACATCTGCGACTAATTTTATTCTTCTTTTTATCATAAAATCTACAGCTAAACTTTTCTCACCTAAATAC<br>ATCTCTGCTTTAATCTACATTTTTGCTTCTAGCACCTAAGTTGGAAGCATTTCCTGAGGCCTCCGGTCAAATCCTGTGTT<br>CTTTTCTAGACTTTGATTGACAGGCCCAATGTCGAGAGAATTCCTTGCATTTTGAATTCCAACAGAATCTCACAGACCCG<br>AGGCTCTCTTGTGACACAGTAGGGCCTGGTGGAACCAAGGGCAGCGTTGTGACACCCCAGAACCTCACGGAACCGAGTGT<br>CAGTCGAGACACAGCGGGGCCTGGGGAAAGGAAATCTCACTGCCAAAGCATTTCTGGCTGATAAAAGTTTGATTTGGGCC<br>AACACCCAGGGATGCTCGAGGGTCTCCATCCCTCTGGGCCTGGGAAATGCTCGAGGTGGCCGGGGGGTTTCTGTCCCTCT<br>CCCTTGGGGGTGGGGTCTTCATCCCTCTGGAGACACCGCAGGGTCTCTGTCCCTCTCACCCGAGGATGAGCGCCCCGAGC<br>ATCCCCCGGTGTGACACGGCCAGAGACCTCCAGCCCTGCCATTCCCCTCAGGTGAGGCCCGAAACCCCCCAGACCGCCCA<br>GGGAGGAGGGGGAACAAAGACATCCCTCGGCCCACCGCGCTCCCTGCCCGGCTGAGGGTCTCTGTCTTCCTCTTCATGGGG<br>CGGGGGGGGCTTTGAGCTTGGTTTGGTTGGCGAGGGACTCGTTGCGAGAGTAAAGACCAGGCACAGCACTTGATGTGATT<br>TTTGCCAATTTTATTTTGGCCTTCGATTTTTTGAAGCGGGACGATGGCCAGACCCCTCCAATTTGGGGGGCCGGCGCGGT<br>CCCTCGCTGCCCCGGTGCTTGTCTGTCCTTAGCTTTGTTGAGCCGGGGTGGTGTCCGACCCCCTCCGATTTTGGGAGGCC<br>GCTGTGCTCTCCATCCACCTTACCTTTGGCAGGCTGTGCTGTCTTTGTCACCTTTTGGCGGGGAGGGGAAAGCGCTGTCG<br>GTGTCCGTCATCCTTGCCCCCGGGGGGAGGCTCACCCCCTGTCCTGGGGTGACGTTGCAGTGCTTTATATCCACAATCGT<br>GTGCTGCTCATGCCTGGAGGGTTTGTTTTGTCTAGCTAGACAGCGCCCCTCCCCATCGGTGAGCTGCTGCCGCTGTGCTG<br>CTTGCAAGCTGCTGCCCCCTCTCCCCCCCTGCGCGGCTCGGCCGCTTTTAGCTGCTTCTGCTTGCTTAGCTGCTGCTTCT<br>GCCCCCCACTTTGCTTAGGCTGCTTCGCTTTATTTTTCCTTTCCTTATTAGCTAGAAATACCACGTGAACCTGATCCTGG<br>ACGACCTGGAACATCAAGAAACGCCGGGCGCCTGCTCGTGAAAAGAAATACGCGTTTCTGCCCTCCCCGGCACGGGAGAT<br>GTCCTGAGGCTGTACATCCACTCGGACGGGTGAAGTTTGCTGCGTCAACCTTGGCTCTTTTTTTCTTGCGTTGGAGAGAG<br>ATTTTTCTGTGTGTTTGCTAAATAAACAAGTGTTTTCGCCTTTTTCTCTGAGGAAAATCTTCCCGAAGCCGGTGGTGGGA<br>GAGGCAAGAGTTTGGAGAGTTTGTTGCTTAAGGGGTTCCTTTTGGAGATTCTTCCCCTAATTTGCCCTAAACCAGGACAC<br>GCCTGTATCTGCCCTCAGCCCAGGGCGATGCTCTGTCCGGCTCCGTCATTGCCCCGGGGAGATGCTCTGTCGGTGTCCGC<br>CCTCCTCGCCCCGAGGGAGGAGCGGCTTGTCTCGCCGTGCCCATCCTTTCTGTGTGGGGATGGTGGGGGTGGTCAGGACA<br>TGTCCGCCTGCTTCCCATTACTGGGAGGGGTCTGGGTGGCCTCCGTGCTTACCTTGGTGTGGGTGGGGGTGGTCTCCATG<br>GAG	0.752	0.862	0.443	717	7393	0.387
CL20	0.063	0.063	143	0.258	1883	TTTTTTGGTTGCTGCTTTGTGAGGAGGAGAAGCCCTGCTCATTTTCTCTTGATGAAGAAAGCAAAAGCATTTTGCTCAAA<br>ATTTCTTCCCCTTTGAAGAAAAGGATGAGATTCTGTTAGTGTTTTCATTTTCCATGTCTGCAGCAGCAACAGCTGGGCAT<br>TTCCTAATTTTCCATTCTAGTGTTTTAAGGAACTCAAAGGTCAGAATTCAGACACAACCTTGCTTGCCGACAGTATCCAT<br>TCTGGAATATTGAATTCAAAGCCACATGCAGTGCTGTTGTAGCAGCCCATCTTAATTTAGTTGGAGTGACTTTGAATCCC<br>ATTGCTATGACCGTTTATGGTTCTGCCTCAGTAGATCAGGTTGTAGAGCTGAATAGAACTGGGATTCCAAATGATAGATA<br>ATGGCTCATTCCTCATGCTTCTCTCTGTCCACAGCGCCCAGAAGCAGCCCCAGACGATGCAGTGACCCTGTCAGCTGCTG<br>GAGATGAAGCCATAGAGGAGGAAAATGACAACAAGCCTCCAGCTATGGTCCCAGCAGAAGCTGAATATTCGGAGATGGTA<br>AGTGCCACTTCTGGCTGGTGCTGTCTTTATTGCAAGAGAGCGCTGCAACTTTCTGAGCAGCCAGCCCTTGCCGTTGGTGG<br>CTGAGGGTGCTGAGTGCGGGAGTCCTGCCAGGAGCTAGCACTCCCTCTTGGCAGTGACAGGTGGAAAATGGGCTTAAATC<br>TGGCATGATTTGGCCGCAGTCTGCTGGGATTGTGATAAGGGCCAGTCTGAAGCACGAAGGCTCTTTGGCCCTAGAGGAGG<br>GAGTGTGTCAATGACACAGTTGTGATGGACACGTAGCAGGACACTGTGGTCTGGGGAGACGTGTCCCACGGCAGAGTCCT<br>GCCAAGGAGTTACAAGCACAAAAATGGGTGATCTGAGCCCACAGTCACTTTGGGGTGCTTTGTCCTCACCAGATGCTTTT<br>CTCTACTTGCCTGGATTCCATTAAGAGACACATTGTGGAGTTGCAGCTTGAAGTGGCAGATTTGGTGTCTTTATTGCCAG<br>GTGGTTCTTTTGCCTCTTCGGCCAGAGCAGTCAGTAGCAGAGTGCAGTCTGAGTGCAGCTGTGTTCTGATAAGGTGCAAA<br>GAGATGATTGTGGTTTCCCTGAATGCTTGTGGGCCTACTCACATCTTATGTGTCAAAACAGCGAAATTGCATACTTGTGG<br>ACCAATTTCAGAGGCAAAATCCAGATGCTACATGAATCCCGGGATACTTTGTGTTGCCTCATGTGCCTTGGTAACTCTGT<br>TTAAAAATGCCATCGAAGCCTTCAGAGGGGAAAAGCTGCACAAGCTTGACTGCAGTCTTGTGTTGCTTAAACGAAGAAGG<br>TTTTGGAAATGCGGATGCTGTATCCAAAGTCAAATGTGCAAGTTTGCATCTGGGGAGCTGCCTGAGACAAGGGGAACCAG<br>TGTTGCCCAACAAGAGCAAGTGAGCAAGAGGCAGCGCGTGCCCAGGTGGCCAAGAAGGCCAAGGGCATCGTGGCGTGTGT<br>CAGCAGCTGTGGGCAGCAGGAGCAGGGCAGTGAGCTGTGCCCTGTGCTGGGCACCGCTGCGGCCGCTGCTCGAGTGCTG<br>TGTCCAGTTGTGGGCCCCTGTGGAGCAGAAAGACATTGAGGGGCTGGAGCGTGTCCAGAGAAGGGCAAGGGAGCTGGGGA<br>GGGGTGGGGGAACAAGCTCAGTGAGGAGGCGCAGAGGGATCTTTAGCCTGGGGAGAGGGAGGCTCATGACGGACATTGCA<br>GGCTCAATTCCAGAAATGCCTACTTGACAGTGCTCCCCACAGGTCTGTTTCTGTGACAAGAATGCCCTCAGTGCATCCA<br>GATGCTTTAAGCATTGGAGTCGGTGACACTTTGATCGTTTGGT	0.916	0.810	0.834	143	729	0.739

Supplementary Table 2. RepeatExplorer2/TAREAN output for eight tandem repeats identified using 200,000 paired-end reads from *Luscinia luscinia*

Putative satellites (high confidence)

Cluster	Proportion[%]	Proportion adjusted[%]	Number of reads	Satellite probability	Consensus length	Consensus	Connected component index C	Pair completeness index P	TAREAN k-mer coverage	|V|	|E|	PBS score
CL1	4.000	4.000	14560	0.993	436	ACATAGTTATTCGTCGTGCAGATTTCTGTGCTAGATATCAGTGATGCAGAGAAGCCATTTCTAGGTGTAGTGCAAAGTGA<br>GATCTGTGGTGTGCTGTGCACACTTTCGCTTTAGCTTTAGGTGCACTGCAGTGCTCTTCGCTATGCATAGTGGAAAGAGT<br>GTATGGTATGCTTTCAAGAAGCAGAGAATATTGCTATGGTAGAGAGGTTTTAGAAGAAAAGTGCACATGCCAGGCGTGTT<br>TTGGCAGTAGCAGAACTTCCCAGCAGAGTGTTCTGTGAGAGGGGTAGACGGCACTTCACCTCCCTCAGCCTGAAATCGCT<br>TCTTGCTACAAACCGCCATTAAGGAAACCACACTGCACTGAGGTGCTCTGCACAGAATACCGGTTGTCAGGGACTAGTGG<br>TATGAAGCAGGATATGCCAGAACCAGCTTAGCTACA	0.995	0.959	0.866	10004	1997982	10.000
CL6	0.560	0.560	2023	0.955	467	CAAAATCTCCAGGAATCTGTACAAAAACCATCTACAATCTGCCAAAATACCAAGGATCGATGGGATAGCATGAGGAAAAA<br>TCCAGGGAAAATCAGCCTACCAAATTGGCACTAAGAAACAAAAAATCCCCTCAAAATCCATATGAAAATGGCCAGAAATA<br>GTCCTAAAATCGACACTGAAACAGCCCAAACCGGTCAAAATTCCAAGCATTATTGGGGAAAACATCAGGAAAAAATCCAG<br>CATAAATTGAACCTTAGCAACCCAGGAAATTGCCTAAAAATTCCCACTAAGAAACACAAAAATCCCCCAGAAATTGCACA<br>AAGAATGGCCAGAAAAACTCCTAATATCTGGCCCAAAACCCAGGGAGTGACCTGGACTTTCTAAGGGAAAAAAGTGGAAA<br>AATAACTGGGCAAACTTCCAAAGGAAATGAGACTTTAAATGGCCCAAAATCCCCCCAAAAAATGGCC	0.977	0.936	0.383	2023	233374	0.000
CL17	0.140	0.140	520	0.956	1994	TGGGTGGTGGCCTGGCGCGAGGGTCTCGATGTGGGGCTGGTGGGTGGTGGTTCTTGTTGGGAGCGGGAGCATTATCAGGC<br>CTGAGTGCGTCGTCATCCGTTGGCAGAAGCCGAGGGACGACGGGGCAAGAGCAAGCTGGATGAGTGAGCTTCGAGGTCAA<br>GAGTGGAACGGCGAGGCAAGAGCGAGCTTGGAGCCGAAGAAGGGAGCACAAGAGGCCCTCTGGGCAAAGGGCCTTAATGG<br>CACGGCCAGGGCACCTTTAAGGTGCCGTGGCTGACTTTGCTTGTGCTGGGCGACTTCCCTGCGTGCAGGGTTGCGTCTTT<br>GTTGTGCTTGGCATCTTGCCATTGTGCTCCTTTGGCTGCATGGAGAGAGGGGCAACCCTGTAACGAGGGTGAGCGCTCCT<br>CTCTCCACACCTGAGGGGGGAAACGGAGCTCCCCTAGCCCCCAGCCCGGCCGCCCTGTAAGCAGGGCAGGGCTTTTCCCC<br>AGCCGTGGCAGCTTTCTGCCTGTTGCTGGCCCCTGGCCAGCGTGACCCCCTGTACTCGGGGCGTGTCACCGCCTTCGTCC<br>CCGTAGCAGCCCTGAAGCGCCTCAGCGCGTCGACGCCCCTGTGCCCTCCCCACGGTGCAGTGCGGTGCGGGTCTGATGCT<br>TTTGTCTCCCACGAAGCAGTAGGGATGGCTCCCTTGAGCCCTTTGGCTGCACGGTACAGGCTCCCTCTTCTGGGACTGTC<br>TTCCCCTCCCTCCCTCCCTCCCTTGTCTTTGCATTAGGCGGTGTGAATGAGCCGAGTGCGTTTGTGCTTTAGCCCCCCTT<br>TAGAAATAGAGCGGTGTAGCTTAGGCTTCCCAGGAGAGGAAGAGGAGCTCCGTGCAGCCACAAGCCAGTCCCCAGAATGC<br>TTGCGTGGACAAAGTGTCTGGCCTCGTCCCCTGCTTCCTGAGGACGGTGCGAAGCCGCTTAACGGTAGCGTGGGCAACGT<br>GACCCGCCTGTAGCCGGGGCCTGGGTCTGGGCCTATGGTGCCCTTTGTGCTGGTGAACTGGCAGCGTGCGGGCCTCTTGC<br>TCTGGGCACGTGTCAGGTACTTGCTTGTCCCCTTGTCCCCAGGAGCAGCCCCAGGTCCCTGTAAGCAGGGCTGGGGAGGC<br>TGCTTTAGCCGGGTAACAGGAACCGCTGCGCCCGGGCCTTTGCCAGGGTGCAGGTTGAAGAAGCGCTGGTGCCACCTCGG<br>CGGCAGCCTCGTTGCGGAGGCTGGAGGGGCTTCTGCAGACGGCCATGTTGCCGTGCTGCGCGGCGGCCGGGCTGCGGCCG<br>GCCCCTAATTGGGCGCGGGCCGGAGCCCGTGCTCCCTGGCGCTTGTTGGGGGGTGCCGGGCTGCGGGGGGCGGCCCCGTA<br>AGCGGGGTTGTGTGCGCCCCCCGGTCTCGGCGGGCGGTTGGAGGGACCACCCCCTGTAATCAGGCGGGTGTCCCTGGCCC<br>TGGCCTTTCAGAGGCGTGGGGCCAGGAGTGGTGGGGCCGGGCGTGTGCCCTCCCTGTCGCCAGGGCTGGGCTCCCGGCCC<br>CTGGGGCCCTTGGGCTTTAGGCCGTGAGTTGGGCTGCTCGACTGTGGAGTCTGGTTCCTGTTGCCGTGGGGTGAAGGCCG<br>AGCTCCGTGGCTCCCTGGGTGTGGACCGGCCTGTAAGCAGACTGTGGGTCTGGGCGCCTTTGTGCTCCCTTCAGCTGGCA<br>GCCTGCGTTGAGCGCCCTCCCCGTTAGTGGGGCGGGGGCCCGAATTGGAGAGAAGGAGCCTCCATAGGCCTTGGGACCAG<br>GGCCGCCCTGTAAGTCGGGGCCGGTGCTCTGGAGTCCCGGGCACTTTGCAGTGCAGGCAGCGGCTTGAGCCCCCTGTAAG<br>CAGGGCTGCAGCCTCTTGTGCGCTGCTTTGCTTTGTGCCCAGCTTCTTTTGCCCAGAGGGCCTGGCTCCCAGCACGGCGT<br>GGGACGTTGGCGGACGTCGAGGTGGTGAGTCAAGCTGGAGCTGGAGGCTTTTGGGCGGCTGCGGGCGGGTCCTG	0.979	0.912	0.849	520	11363	0.066
CL49	0.023	0.023	85	0.741	1181	CTCCAAGAGCCTTCAGCCCTGCCAGAGCCTTGGTGAGAGCTGCCAGAACGCAGTGGGAGGCAGCCCTTGGGACAGGCATC<br>CCTGCTCCCTCAGAGTCACGGCTATGGGACAAACACCCTCATCCCTGAGGCTGCAGATGGGCAGGAAAAGCCCAAAAGCA<br>AAGCTGGGAGTGCAGCCCACACGAGTAAGGCAATGCCAGAAGAATGTGTCCCAGGAGCAAATATGCTGGGGCTGCCAATT<br>GCCAGTGCATGGCTCTGAGGAAAATGCAGTTGTGGCATGGGAGAAGGGAGAGCCTTGGTGTGGTGCCGGACAACGATCCT<br>CCAGCAGCAAAGGCAGCAGCGTTCCCATGTGCAGCTGAGTCTCTGGAGCAGGCAGTGCAGGAGCAGGAAGAGCAAGATCT<br>CCACAACTGGATGACTTCTGGCAAGAGTCTCCATAGCTCCCGGATCCTTTGTGATAGCTGCCGGACTGGAGAGAGAGAGG<br>AAGCCCTTTGGACAGGACTGCCTGCACCCTCAGGTCCGTGGGCATCTGTAAAGTAGGAATGATTCACATACTCAGTCTTC<br>ATACATGCAGGAGTCCTTCTTACAAATGTACATAGATTTAATAACTTGCTGGCTATCAGGTTAGAATAGCTTGAGCCTGG<br>ATAAGCAATAGTATAAATGCTCAGCTGTCAGCTAAGCAATAACATACATGGTCAATCCCCTAGTAGCTACATCAGATTTT<br>TTTTCTTAAGACAAGTGCTTGTAGAACAATTCCATCTTCAAACGACCTTACTTCTGCTACTTAGTCCATTTTAGAACCCT<br>TTTTAGAAATCACATGCCTTAACTGAATAGCGTTCCTTGTTCTCTTATTCCAAATTGTCTTTGTCTTTTTTCCCCACAGG<br>TATCGGCAGAACCTGCTCATCCCTGAGCATGCACAATTGCCAGGGAAATCCAGAAAGCAAACCTGGAGGCCAAGGCCGTT<br>CCAGAAGGTGTTTCCTGAAGCCAGACACACGGCAGCAATTTCCAGAGCTGTCACTTCCTCGGCCACAGCGTTACGCATGC<br>TCTGGCTGTGCCGCAGGACAAGAGCCTTGGTGTTGTCCTGGAACAGGTTCCTGCAGAAGAAAAGGTGGTGTTCCTGTGTG<br>CAGCGAACCCTTTGGCATCAGCAGTAGAGGAGCACAATGCTGAAGATCTCCAGCCTTTTTT	0.953	0.889	0.880	85	505	0.442

Putative satellites (low confidence)

Cluster	Proportion[%]	Proportion adjusted[%]	Number of reads	Satellite probability	Consensus length	Consensus	Connected component index C	Pair completeness index P	TAREAN k-mer coverage	|V|	|E|	PBS score
CL23	0.067	0.067	245	0.3930	1881	ACCAAACCATCAAAGTGTCACCGACTCCAATGCTTAAAGCATCTGGATGCACTGAGGGCATTCTTGTCACAGAAACAGAC<br>CCTCTGGGGAGCACGGTCAAGTAGGTATTTCTGGAATTGTGCCTGCAATGTCCATCCTGCGTCTCCCTCTCCCAAGGCTA<br>AAGATCCCTCTGCGCCTCCTCACTGAGCTTGCTCCCCACCCTCCCCAGCTCCCTTGCCCTTCTCCAGACACCATCAACC<br>CCTCAATGGCTTTCTGCTCCACAGGGGCCCACAACTGGACACAGCACTCGAGCAGCGGCCGCAGCGGTGCCCAGCACAGG<br>GCACAGCTCACTGCCCTGCTCCTGCTGCCCCACTGCTGCTGACACACGCCACGATGCCCTTGGCCTTCTTGGCCACCTGG<br>GCACGCGCTGCCTCTTGCTCACTTGCTCTTGTTGGGCACCACTGGTCCCCTTGTCTCAGGCAGCTTCCCAGCACGCAAAC<br>TTGCACATTTGACTTTGGATACAGCATCCGCATTTCCAAAACCTTCTTGGTTTTAGCAACACAAGACTGCAATCAAGCTT<br>GTGCTGCTTTTCCCCCTCTGCAGGCTTCCACGGCATTTTTAAACAGTTACCAAGGCACATGAGGCAACACAAAGTATCTC<br>GGGATTCATGTAGCATCTGGATTTTCTCTCTGAAATTGGTTTACAGGTATGCAGTTTGGCTGTTTTAACACATGAGATGT<br>GAGTAGGCCCACAAGGATTCATGGACACCACAATCATCTCTTTGCACCTTATCAGAACACAGCTGCACTCACACTGCACT<br>CTGCTACTGACCGCTCTGCCCGAAAAGGCAAAAGAACCACCTGGCAATAAAGACACCAAATCTGCCACTTCAAGTTGCAA<br>CTCCACAATGTGTCTCTTAAAGGAATCCAGGCAAGTATAGAAAAGCATCTGGTAAGGATAAAGCACCCCAAAGTGACTGT<br>GGGCTCAGATCACCCATTTTGTGCTTGTACCTCCTTGGCAGGACTCTGCAGTGGGACACGTCTCCCCAGACCACAGTGT<br>CCTGCTACGTGTCCATCACAACTGTGTCATTGACACACTCCCTCCTCTAGGGCCAGGGAGCCTTCGTGCTTCAGACTGGC<br>CCTTATCACAATCCCAGCAGACTGCGGCCAAATCATGCCAGATTTAAGCCCGTTTTCCACCTGTCACTGCCAAGAGGAG<br>TGCTAGCTCCTGGCAGGACTCCTGCACTCAGCACCCTCAGCCACCAACGGCAAGGGCTGGCTGCTCAGAAAGTTGCAGCG<br>CTCTCTTGCAAGAAAGACAGCACCAGCCAGAAGTGGCACTTACCGTCCCCGAATATTCAGCTTCTCCTGGGACCATAGCT<br>GGAGGCTTGTTGTCATTTCCTCCTCTATGGCTTCATCTCCAGCAGCTGACAGGGTCACTGCATCGTCTGGGGCTGCTTC<br>TGGGCACTGTGGACAGAGAGAAGCATGAGGGATGAGCCATTATCTATCATTTGGGATCCCAGTTCTATTCAGCTCTACAA<br>CCTGATCTACTGAGGCAGAACCATAAACGGTCAGAGCAATGGGATTCAAAGTCACGCCAACGAAATTAAGATGGACTGCT<br>ACAACAGCACTGCATGTGGCTTTGAATTCAATATTCCAGAATGGATACTGTCAGCAAGCAAGGTTCTGCCTGAATACTGA<br>CCTTTGAGTTCCTTAAAGCTCTAAAATGGAAAATTAGGAAATGCCCAGCTGTTGCTGCTGCAGCATGGAAAATGAAAAC<br>ACTAACAGAATCTCATCCTTTCTTCAAAAGGGAAGAAATTTTGAGCAAAATGCTTTTGCTTTCTTCATCAAGAGAAAAT<br>GAGCAAGGCTTCTCCTTCTCACAATGAAGCCACGGAAAAAA	0.922	0.899	0.848	245	2277	0.686
CL28	0.054	0.054	195	0.2420	63	CGCCACGGCCGGCCCACCCGTGGGTGGGCGTGCAGAACTGTTTGCGTGTGCCACTGGGTGTGC	0.954	0.789	0.630	195	11447	0.000
CL32	0.039	0.039	140	0.0147	756	TTGAATGACATCACCCAGTCTTAACTGACCTTATGAATGTGGGGACGGGATTTTGTTTCACAGGTTAAAGGGCCACTAT<br>TCATGTCCAGTGCCCCGGGCCTGAGAATTAAGTCCGCCTGCTAAAATGAGAAACAAATGAATATTTCTCCCAGATGCAAG<br>GTGCCATCACTTACATCTATGTCTGCCTGCAGAGTGACAAAGGCCTTCTCCAAACTGGTATCGGGAAACTGGGAGGGTGA<br>GTATTTTTTCAAAGAGAAATAAGGCCATTATTTGTGTCCAGGGCCCTGTGGCTGAGACCGGGCTTCTGCATGCTGAAATT<br>CTGAACGGATCAGCATTTCTCCCAGACACAAACTGACACCACTTAGAACCCTGGCTGCTTTTGAGTGCCAAAAGCCTCC<br>TGTGAATAGACATTACAAAAGTGGTGAAGAGAGCTGTATTTGACAGAGAAATGGGCTCGTTTCTGTCCATTGCCCAGTGA<br>ATGAAACGGGGCTTCTGCCTGCTAAAATTCAAACAGTTCAGGATGTCTCACACACAATCTGGCAACACTTAGAACCCTG<br>GCTGTTCTTTGAATTCCAAACACCCGCTCTAAACTGACATTCCAAAAGTGGTGAAGTGAGCTGTATTTCACAGAGAAATA<br>GGCCCCTGTTTGTGTCCATTACCCAATGAATGAAGTGAGGCTTCTGCCTGCTAAAACTCCAAACTGTTTGGAAATCTTGC<br>AGATAGAAACGGGCACCATTAGAACCCTGGCTGTTC	0.714	0.818	0.466	140	741	1.170
CL59	0.018	0.018	65	0.4210	80	CAAAAAATCCCTTCCTAATCTCTGGATTCATGGGGGAGCATTGAAAAAAAGCTGGGATTAAAGGAGCAACTGAGCCAACT	0.969	0.806	0.528	65	603	0.000
